# Benefits and Costs of Digital Consulting in Clinics Serving Young People With Long-Term Conditions: Mixed-Methods Approach

**DOI:** 10.2196/medinform.9577

**Published:** 2018-10-30

**Authors:** Sung Wook Kim, Jason Madan, Melina Dritsaki, Carol Bryce, Vera Forjaz, Joe Fraser, Frances Griffiths, Kathryn Hamilton, Caroline Huxley, Jackie Sturt

**Affiliations:** 1 Division of Health Sciences Warwick Medical School The University of Warwick Coventry United Kingdom; 2 Oxford Clinical Trials Research Unit, Botnar Research Centre Nuffield Department of Orthopaedics, Rheumatology and Musculoskeletal Sciences University of Oxford Oxford United Kingdom; 3 Department of Clinical, Educational and Health Psychology University College London London United Kingdom; 4 Joe's Diabetes Ltd Coventry United Kingdom

**Keywords:** digital consulting, long-term conditions, costing analysis, young people, mental health, diabetes

## Abstract

**Background:**

Since the introduction of digital health technologies in National Health Service (NHS), health professionals are starting to use email, text, and other digital methods to consult with their patients in a timely manner. There is lack of evidence regarding the economic impact of digital consulting in the United Kingdom (UK) NHS.

**Objective:**

This study aimed to estimate the direct costs associated with digital consulting as an adjunct to routine care at 18 clinics serving young people aged 16-24 years with long-term conditions.

**Methods:**

This study uses both quantitative and qualitative approaches. Semistructured interviews were conducted with 173 clinical team members on the impacts of digital consulting. A structured questionnaire was developed and used for 115 health professionals across 12 health conditions at 18 sites in the United Kingdom to collect data on time and other resources used for digital consulting. A follow-up semistructured interview was conducted with a single senior clinician at each site to clarify the mechanisms through which digital consulting use might lead to outcomes relevant to economic evaluation. We used the two-part model to see the association between the time spent on digital consulting and the job role of staff, type of clinic, and the average length of the working hours using digital consulting.

**Results:**

When estimated using the two-part model, consultants spent less time on digital consulting compared with nurses (95.48 minutes; *P*<.001), physiotherapists (55.3 minutes; *P*<.001), and psychologists (31.67 minutes; *P*<.001). Part-time staff spent less time using digital consulting than full-time staff despite insignificant result (*P*=.15). Time spent on digital consulting differed across sites, and no clear pattern in using digital consulting was found. Health professionals qualitatively identified the following 4 potential economic impacts for the NHS: decreasing adverse events, improving patient well-being, decreasing wait lists, and staff workload. We did not find evidence to suggest that the clinical condition was associated with digital consulting use.

**Conclusions:**

Nurses and physiotherapists were the greatest users of digital consulting. Teams appear to use an efficient triage system with the most expensive members digitally consulting less than lower-paid team members. Staff report showed concerns regarding time spent digitally consulting, which implies that direct costs increase. There remain considerable gaps in evidence related to cost-effectiveness of digital consulting, but this study has highlighted important cost-related outcomes for assessment in future cost-effectiveness trials of digital consulting.

## Introduction

Improving efficiency in the National Health Service (NHS), particularly regarding managing long-term conditions, is a major policy goal in the United Kingdom (UK). NHS aims to secure the greatest improvement in the health of the people living in England under 3 themes: equity, efficiency, and responsiveness [1]. Efficiency is sometimes misinterpreted as involving cost savings and reduced budgets, but it actually refers to maximizing the value generated by whatever resources are available to NHS in terms of the quantity and quality of health care it provides [2]. The use of digital communication for consultation, such as email, text, and mobile, has been proposed as a way of enhancing NHS efficiency [3]. Care home residents in West Yorkshire, for example, were offered a service that connects them with clinicians using a video link [4]. By consulting digitally, it is possible to ensure equitable access to NHS care for people living in geographically isolated areas [5]. Under this current trend, the use of digital consulting is expected to increase in the United Kingdom over the next few years. A particular area of interest for applying digital consulting is the management of individuals with long-term chronic conditions. Such individuals account for 80% of consultations, and 10% of patients with long-term conditions account for 55% of inpatient days [6]. The cost of inpatient days for patients with long-term conditions are expected to be a significant cost burden for NHS. In addition to this, managing long-term conditions is expensive for NHS with treatment costs accounting for approximately 69% of all health care costs in England in 2008 [7]. Given these facts, the potential gains from improving the efficiency of care for people with long-term conditions are enormous [8].

Digital communication has been assumed to be a route for delivering quality care at lower cost. However, this is not necessarily reflective of experience with health investment in information technology to date. The Watcher report warns against such assumptions, particularly over short-term horizons, and argues that financial savings may take 10 years to be realized [9]. This applies to patient-health professional digital consulting because the costs of implementing it are unknown. It is not inevitable that using email and text as examples of digital consulting will reduce the workload of clinics. It was argued that growth in email communication with patients has increased the workload of clinicians because they need to respond to more patients [10].

In terms of the impact of digital clinical communication on both patients and service providers, the evidence is somehow conflicting. One cancer trial study reported that a higher level of use for a Web-based health support system is associated with improved outcomes, such as mood and quality of life [11]. Email consultation can improve continuity of care and thus is likely to improve the quality of care and that of life [12]. Other potential benefits of digital communications include improvement in health care management and an improved patient-doctor relationship [13]. The smartphone is likely to make behavioral health therapy more interactive for patients, improving the delivery of evidence-based medicine [14]. A systematic review found that the clinical outcomes such as the HbA_1c_ level for diabetic patients and forced expiratory volume for asthmatic patients were improved when asynchronous communication such as short message service (SMS) text message was used [15]. Other studies [16,17] suggest that there is no difference between digital and usual care. Bradford et al [16] reported that there was no difference in the quality of life scores between caregivers in control and intervention groups who used a home telehealth service for pediatric palliative care. It was also reported that offering treatment using Web-based consultations for child dermatitis was not effective compared with traditional treatment, such as visiting general practitioners [17]. These examples show that there is no clear agreement in the effectiveness of consulting based on digital communication.

Although digital consulting between clinician and patient is of general interest in NHS, few studies have considered cost. Much of the research has focused only on the “effectiveness” of digital communication [18-20]. This may be because the direct costs of services of using text, mobile, or internet communication appear low [21,22]. Moreover, two systematic reviews on networked communications found that telemedicine cost is cheaper than travel costs [23,24]. Nevertheless, the impact of digital consulting on staff workload is unknown. It is possible that digital consulting will reduce the time involved in each consultation, but an alternative possibility is that it increases the volume of communication and hence increases staff workload without adding significant value to patient care. This information is important as time spent on digital consulting, if substantial, could have a significant impact on NHS.

The purpose of this study was to explore the economic impact of digital clinical consulting that occurs using email, text, mobile phone, and Web portals with young people having long-term conditions as part of the LYNC study [25,26]. The LYNC study explored how health care delivery and receipt are impacted by the use of digital consulting for patients and health professionals. To study the health economic impacts, we used a mixed-methods approach to address the following questions: What are the main drivers of the time spent on digital communication? What are the health benefits of digital communication as perceived by patients? Are there any benefits of digital communication beyond health?

## Methods

The LYNC study focused on young people with long-term health conditions because they are more likely to disengage from health services and be associated with additional cost for NHS. The Long-term conditions Young people Networked Communication (LYNC) study attempted to look into whether their engagement can be improved using digital consulting. The researchers working in the LYNC study both qualitatively and quantitatively interviewed clinical staff to understand how digital consulting is used to communicate with young people. The duration of this study was approximately 3.5 years. The LYNC study was an observational mixed-methods study that aimed to identify the effects, impacts, costs, patient safety, and ethical implications of digital consulting using email, text, social media, and personnel health records between health care professionals and young people living with 1 of 13 different physical or mental long-term health conditions [25].

We considered mobile phone calls differently from other phone calls because they could be initiated by young people when they needed, wherever they were located. The use of mobile phones for calls was important to some young people because it is often difficult to connect with a clinician through standard telephone systems within the UK NHS systems. The rationale for considering mobile phone calls as digital relates to the portability of mobile phones, which changes the relationship between the young person and the communication such that they have control and flexibility over the communication that took place.

Mobile phones were also valued as a communication route by clinical teams because they often found it difficult to reach young people on landlines, given that they were not often there during clinicians’ working hours and may not answer the landline or respond to the messages left.

The study involved a total of 173 clinical team members, including clinicians, psychologists, psychiatrists, dieticians, and nurses, 165 young people living with long-term conditions, 13 parents, and 16 information governance specialists from 20 clinics across the UK NHS between November 2014 and March 2016. Clinics were eligible for inclusion if they provided specialist care for young people (age 16-24 years) with long-term conditions and if the clinical team had an interest in using digital consulting as part of the services provided to patients. Ethical approval for the LYNC study (14/WM/0066) was obtained from the National Research Ethics Service Committee West Midlands—The Black Country.

A health economic questionnaire (Multimedia Appendix 1) was designed with the purpose of collecting information from clinical staff members with respect to their use of digital consulting in the clinic. This questionnaire was developed following, and drawing on insights from, the initial 115 semistructured interviews conducted for the main LYNC study. The LYNC participant was asked to complete the questionnaire during the interview wherever possible, although in some cases, it was completed postinterview and returned by email. This questionnaire elicited information about the time spent using digital consulting to communicate with young patients, type of communication being used (eg, email, mobile phone calls, text, or other), staff grade, and the number of hours the staff member worked per week. Clinical staff members were asked to recollect their approximate time spent using digital consulting with patients per week in time intervals (eg, 15-30 minutes and 45-60 minutes). They were also asked about the equipment they used to communicate via digital consulting (eg, laptop, desktop, tablet, or mobile phone).

The time spent digitally consulting, as reported by clinical staff, was costed using the midpoint salary for their grade based on the NHS Agenda for Change 2014/2015 salary scales [27]. In the UK NHS system, “grade” is equivalent to “band” [28]. Grade covers all NHS staff other than doctors, dentists, and senior managers, and the 9 pay bands have multiple pay points [27]. Therefore, we used “grade” to estimate staff salary per hour.

Equipment costs were estimated using price lists provided by the University of Warwick. These costs were annualized assuming a 3.5% discount rate based on methods guidance from National Institute for Health and Care Excellence [29]. For clinical sites with questionnaire completion rates over 50%, estimates of clinic list size were requested to allow estimation of cost per patient. A health service perspective was adopted for the costing analysis in this study. In fact, the LYNC study collected health economic data from various sites with long-term conditions rather than focusing on only one disease site. Hence, the costing analysis in this study covers a wide range of costs. Among them, we could not get sufficient data from 4 sites—Dermatology, Mental health 3, Sickle cell, and Diabetes 2. This is because sites were counted only if their overall completion rate was over 50%. If less than 50% of the questionnaires were completed at a site, we did not attempt to calculate clinic-level costs.

A regression analysis was carried out to estimate the main drivers for the time spent on digital consulting activity. A two-part model [30] was employed to analyze the data collected from the clinical staff to identify any factors associated with the time spent on digital consulting. This model was chosen to reflect the fact that the time spent on using digital consulting is rightly skewed and contains many zeros [30]. The logit model was used for the first part, whereas the generalized linear model with the Poisson family was used for the second part after conducting the modified links test [31]. Log link was used in this regression. All regression analyses were carried out using Stata 14 (Statacorp, College Station, TX, US).

Mechanisms through which digital consulting may affect outcomes relevant to economic evaluation, for patients and services, was derived through the initial semistructured interviews of clinical staff. One health economist (SWK) read all interview transcriptions from staff to identify statements that reflected a consequence of digital consulting use that might lead to effects pertinent to economic evaluation. Relevant quotes were extracted to populate a thematic analysis of the purpose and content of digital consulting between clinical staff and patients; specific examples of how it was being used; the results of using digital consulting; the counterfactual showing what would have occurred if digital consulting was not available in this situation; and the incremental consequences in terms of costs and benefits to patients (whether health related). To ensure that extraction was consistent and appropriate, a sample of the transcripts from each site was independently reviewed by two other health economists (MD and JM) who then provided feedback to the primary health economic reviewer. Further information on the clinical implications of digital consulting use identified in the transcripts was provided by clinical experts in the LYNC study team.

It was rarely possible to determine all relevant information for the economic thematic analysis from the initial interviews. Therefore, once gaps in any of the domains had been identified, follow-up interviews were conducted by one health economist (MD) with clinical leads at each of the sites to expand on the information initially identified. These interviews were structured based on a bespoke interview guide developed for each site by the health economics team based on identified gaps in the thematic analysis.

## Results

### Quantitative Findings

A total of 115 staff (66.5%; 115/173) supplied health economic questionnaire data from 18 clinical sites. Table 1 provides a descriptive summary of these responses, broken down by channel, site, and role.

Email (mean=30 minutes; interquartile range [IQR]=0-45) and mobile (mean=20 minutes; IQR=0-30) were preferred methods for digital consulting. Staff reported that they use social media less (mean=3 minutes; IQR=0) than other channels.

**Table 1:** Breakdown of digital consulting use by channel, site, and staff role (minutes per day).

Breakdown of use		N	Mean	Interquartile range	
**Channel**					
	Email	115	30		0-45
	Text	115	17		0-15
	Social media	115	3		0-0
	Mobile	115	20		0-30
**Site**					
	Rheumatology	8	73		23-105
	Mental health 2	6	8		0-15
	Mental health 1	8	184		98-225
	Renal	6	7		0-10
	Dermatology	2	28		25-30
	Diabetes 1	6	53		45-60
	Sickle cell	6	133		45-180
	Mental health 3	4	21		13-30
	Liver	7	104		15-120
	Inflammatory bowel disease 2	4	105		0-210
	Cystic fibrosis 1	11	56		0-120
	Diabetes 2	2	8		0-15
	Cystic fibrosis 2	3	90		0-150
	Sexual health	10	107		0-165
	HIV	9	26		0-45
	Cancer 2	11	97		21-150
	Inflammatory bowel disease 1	7	64		0-120
	Cancer 1	5	86		10-165
**Role**					
	Physiotherapist	4	120		83-158
	Psychologist	11	34		0-30
	Dietician	8	14		0-15
	Nurse	31	120		30-180
	Consultant	31	28		0-45
	Other	30	105		5-165

The time spent on using digital consulting was highest at the Mental health 1 site (mean=184 minutes; IQR=98-225) and lowest at the Renal site (mean=7 minutes; IQR=0-10). Staff in the Mental health 2 site (mean=8; IQR=0-15) and Diabetes 2 site (mean=8; IQR=0-15) also spent less time on digital consulting than staff in other sites.

Nurses (mean=120 minutes) and physiotherapists (mean=120 minutes; IQR=83-158) used digital consulting for approximately 2 hours; however, dieticians (mean=14 minutes; IQR=0-15) and psychologists (mean=34 minutes; IQR=0-30) used less. Psychologists reported that they use digital consulting less (mean=34 minutes; IQR=0-30) than nurses or physiotherapists.

**Figure 1:**
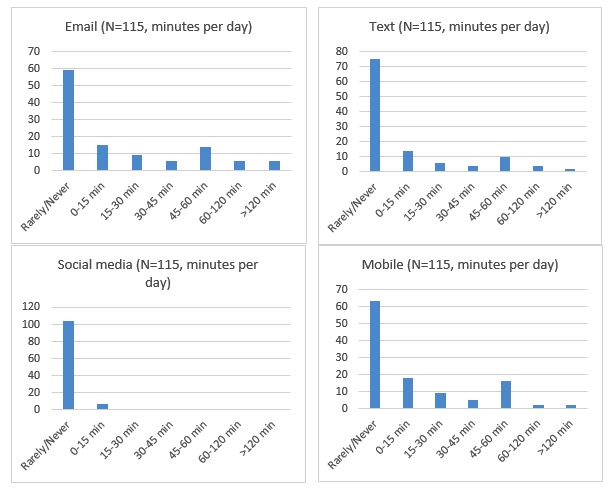
Breakdown of time spent by staff in digital consulting with young people (minutes per day) by channel.

The type of digital consulting used for clinical communication is illustrated in Figure 1. With respect to text, 34.8% (40/115) of the staff reported that they use a text, and 15% (6/40) of them mentioned that the time for using text as part of communicating with patients accounted for over 60 minutes per day. On the other hand, social media had rarely been used, that is, 9.6% (11/115) of staff replied that they use social media to communicate with patients. This result suggests that the use of digital consulting is mainly concentrated on email, SMS text messages and mobile phone than social media. One clinic (Rheumatology) did not use digital consulting at all, and a second clinic (Renal) reported just 7 minutes of use per day. The heaviest user consulted digitally with patients for more than 2 hours per day.

A clinic-level costing analysis was carried out to estimate the direct burden of digital consulting to the clinic, and the result is presented in the main clinical paper of the LYNC project [25]. The total cost was highest at one of the two Child and Adolescent Mental Health Services (£9560) and lowest at the Renal site (£161) except for the Rheumatology site (£0). The interviewed staff in the Rheumatology site reported that they never use digital consulting to communicate with patients. The total cost of using digital consulting per staff member ranges from £27 (Renal) to £1195 (Mental health 1) per month. The average cost per patient was particularly high at the Cystic fibrosis sites. The average cost per patient was £130 and £73 at the Cystic fibrosis 2 site and Cystic fibrosis 1 site, respectively. The next highest cost per patient was £16 per month (Renal clinic). The data suggest substantial variation in digital consulting use and therefore accounts for sites managing patients with the same condition. For instance, 2 Mental health sites showed a significant difference in the total cost between sites (£9560 vs £230); likewise, the total cost was different at the Cystic fibrosis (£5706 vs £1559) and Cancer (£3017 vs £6357) sites.

**Table 2:** Factors associated with time spent on using digital consulting: Two-part model analysis (N=81).

Job role^a^		Coefficients from the two-part model (SE)	*P* value
Psychologist		31.67 (9.88)	<001
Physiotherapist		55.3 (14.66)	<001
Dietician		6.54 (6.39)	.31
Nurse		95.48 (9.85)	<001
Other		67.49 (10.20)	<001
Full time		38.49 (26.44)	.15
**Site^b^**			
	Mental health 1	43.36 (24.16)	.07
	Renal	−1.96 (8.25)	.81
	Dermatology	−1.02 (2.51)	.68
	Diabetes 1	11.18 (6.63)	.09
	Sickle cell	37.91 (21.13)	.07
	Mental health 3	28.77 (9.14)	<001
	Liver	100.97 (10.77)	<001
	Inflammatory bowel disease 2	74.89 (33.89)	.03
	Cystic fibrosis 1	16.15 (10.67)	.13
	Diabetes 2	18.84 (14.34)	.19
	Cystic fibrosis 2	18.89 (24.34)	.44
	Sexual health	65.91 (24.07)	.01
	HIV	10.38 (9.95)	.30
	Cancer 2	74.86 (9.56)	<001
	Inflammatory bowel disease 1	74.92 (13.19)	<001
	Cancer 1	80.98 (14.85)	<001

^a^Reference case: consultant.

^b^Reference case: Mental health 2 site.

Regression analysis (Table 2) showed that consultants used digital consulting less compared with other groups. All other groups spent more time using digital consulting (*P*<.001) compared with dieticians (*P*=.31) using a reference case of “Consultant.” Nurses, physiotherapists, and psychologists used digital consulting more than consultants by 95.48, 55.3, and 31.67 minutes, respectively. Full-time staff spent more time using digital consulting than part-time staff, but the result was not statistically significant (*P*=.15). Time spent using digital consulting varied across sites, and no clear tendency in using digital consulting was found. Overall, these results show that being a consultant was a significant (negative) predictor of time spent on digital consulting.

### Qualitative Findings

The qualitative component of the LYNC study [25] provided evidence of mechanisms through which digital communication could generate health benefits. The standard qualitative interviews, based on initial findings on health benefits of digital consultation to young people, identified some mechanisms through which these benefits could be interpreted as being cost-beneficial to the health care system, clinics, and individual patients. We also anticipated these potential economic benefits of digital consulting to signpost a number of important outcomes for health economic assessment in future cost-effectiveness trials.

### Potential Routes to Economic & Clinical Impact

#### Prevention of Adverse Events

Staff and young people reported several examples of how the use of digital consulting enabled services to identify crises early by providing a channel for communication between appointments:

It saved my life basically. And it’s made life more bearable for me not to do anything [self-harm], so...It’s made me open up more to my therapist and stuff, and people on the team. I need to know that they’re there and I can speak to them if I need it. Mental health 3 (outreach team) site.

Young person 01

This allowed for timelier management of adverse events such as self-harm associated with mental health, which could lead to improved health outcomes thus potentially preventing the health care provider from spending more costs on patients’ management.

#### Improved Well-Being

Young people reported psychological benefits from using digital consulting beyond its direct impact on their health. The use of digital consulting can reduce the stress of accessing clinical staff given that a SMS text message can be left at any time day or night, which was considered beneficial by some young people. For other young people, the use of digital consulting improved their access to social and emotional support or social services, which they thought contributed to reduced anxiety. The convenience of using email to contact a busy clinician is articulated by this young person:

So there’s been emailing with the like dietician to see if it’s okay to sort of start back on that. Within the week definitely, like it will never be more than like a couple of days to wait for a reply.

Diabetes 1 Young person 13

In addition to getting a quick reply is the importance of being able to ask questions between standard appointments, “...then I’m not, like, waiting six months to ask whatever questions I have. [Arthritis Young person 13]"

In other words, asynchronicity eased communication between health professionals and patients, which led to young people being able to undertake timely self-care activities which resulted in improved their well-being.

#### Time and Cost Savings for Users

Service users reported that digital consulting saved time and reduced their costs, for example, by reducing their visits to an accident and emergency department “I would probably still come over to A&E as I have done many years before if I did not have their mobile phones numbers. [Sickle cell Young person 08]” or through reducing the number of clinical visits, which was particularly important for those traveling long distances:

Yeah, it would mean that I don’t have to come into clinic all the time. Save parking, save traffic, all that sort of thing.

Liver Young person 18

These statements provide specific examples of how digital consulting can be time- and cost-saving for young people.

### Benefits of Using Digital Consulting for the Clinics

#### Improving Efficiency

It was identified that the use of digital consulting can improve efficiencies from the NHS perspective. Interviewees from a number of sites mentioned that digital consulting is commonly used to book an appointment, and interviewees from a few sites mentioned that the use of digital consulting can reduce missed appointments. The ability to replace some clinic visits, as described above, has benefits for services as well as users. A senior clinician at a diabetes clinic estimated that digital consulting saved approximately 8-12 visits per service user per year across a clinic of 686 patients.

#### Effect on Workload

Although many of the staff interviewed reported that using digital consulting increased their workload, this was not necessarily viewed negatively because they also felt that it improved patient care. Several sites reported that users were more likely to contact the service via digital consulting where they previously would have waited until the next clinic appointment. Digital communications can help young people who would otherwise be lost to the service. Some young people do not like synchronous communication but were willing to communicate asynchronously using digital technology.

Clinical staff saw the value of offering alternative communication pathways for some patients. It was seen as an important two-way communication mechanism to remind young persons that they had not been forgotten and that it is okay for them to communicate in any way they felt comfortable with.

So it's just a little, hi, you know, you're on our mind even though you're not on the ward, kind of thing, to keep that continuation of care going. They like that.

Cancer 2 Support worker 05

This was primarily a consequence of digital consulting enabling ease of access and therefore generating user expectations around staff availability and timeliness of response.

## Discussion

To the best of our knowledge, this study is the first study to employ both qualitative and quantitative approaches to evaluate the benefits and costs of digital consulting in the UK NHS. Using this hybrid approach, it could capture the perceived benefits of using digital consulting for both clinics and patients. The findings of this study provide insights into the likely impact of digital consulting use across various long-term conditions in the UK NHS for young patients.

This study found significant variation in the time spent on digital consulting. Nursing staff were the greatest users of digital communication with the highest costs associated with their working time. It was also found that staff time is likely to be the key driver of the immediate cost impact of digital consulting. Staff time spent on digital consulting varied widely among health care workers with consultants spending less time on digital consulting to communicate with patients compared with other clinical staff members.

There are limitations that need to be noted in this study. First, the time spent on digital consulting is based on the self-reported questionnaires. In other words, this is based on self-estimation of the time spent by the clinical team members rather than the actual measurement of time spent at each site. As a result, the analysis result may be associated with measurement bias for the time spent on digital consulting. We suspect that clinical staff could have underestimated the actual time spent on digital consulting because they may not recall all their activities involving digital consulting methods. To overcome this issue, we would recommend the use of diaries or time logs for their daily activities; however, this is inherently challenging and was beyond the scope of the LYNC study.

Second, the study design did not permit a formal economic evaluation of any specific mode of digital consulting. The requirements of a formal economic evaluation are well established and include the need for clearly defined, mutually exclusive interventions with robust incremental estimates of cost and outcome data [32]. Given the design of the LYNC study, it was simply not possible to conduct a conventional economic analysis of this sort. As a result, although we were able to qualitatively establish the key mechanisms through which costs and benefits result from digital consulting, we were unable to measure the net impact of digital consulting. Specifically, we were unable to quantify the incremental impact of digital consulting use on clinic costs, adverse event rates, long-term disease progression, or “did not attend” rates. These limitations result from the breadth of the study and the fact that it was cross-sectional in nature. Nevertheless, we attempted to supplement these limitations by employing a qualitative approach. The qualitative approach identified staff reports showing that digital consulting increased workload. Also, it was found that digital consulting could lead to benefits and cost savings through qualitative mechanisms.

The qualitative evidence shows that young people value improved access to clinical staff afforded by digital communications and that health professionals viewed themselves to have kept better contact with young people in general and that it was of particular value to reach out to those who found engagement through traditional modes of communication more difficult. This is mainly because digital consulting enables young people to engage in ongoing care with health service providers between appointments, which could lead to improved outcomes for young people and prevent any potential side effects such as self-harm [25]. Mental health patients can control their desire to hurt themselves by contacting clinical staff in time with digital consulting when they feel the urge to inflict self-harm. Digital consulting was also able to offer detailed and personalized information so that young people could receive more responsive care. This suggests that evaluations of digital consulting need to carefully identify and measure its impact on both ongoing management of chronic conditions and the incidence of side effects.

These findings imply that specific digital consulting interventions have the potential to be highly cost-effective. Long-term benefits of using digital consulting may well outweigh the cost of using digital consulting per patient if it is possible to prevent complications and it is not unreasonable to hypothesize that digital consulting may be a cost-effective intervention for promoting patient activation [33] with self-management and reducing complications or secondary comorbidities.

However, two important points should be considered for digital consulting interventions to be cost-effective. First, it should be noted that costs and cost savings occur in different budgets; therefore, costs to the clinic, such as staff workload, may increase, whereas cost savings accrue elsewhere in NHS. Evaluations and service implementations need to carefully assess the impact of digital consultation on staff workload and consider how this impact can be managed and potentially minimized. Second, there are important benefits to digital consulting that go beyond health and into well-being, such as a sense of control over the condition [34]. Young people generally struggle in managing their chronic conditions, facing health and lifestyle impacts beyond their control [35]. For instance, our qualitative analysis revealed that the ease of access to clinical staff, which cannot be directly captured by health-related quality of life outcomes such as quality adjusted life years, can be improved by constant contacting via digital consulting with clinical staff. Moreover, young people find digital consulting less burdensome than face-to-face consultation or direct phone calling to the clinic and therefore contact clinical staff digitally. Self-determination theory suggests that individuals require competence, autonomy, and meaningful relationships to underpin the development of intrinsic motivation [36]. Self-determination may be supported through digital consulting, and this may be the mechanism through which it impacts well-being and quality of life [37]. Consequently, evaluations need to carefully consider whether psychological outcomes that cannot be captured by quality adjusted life years are an important part of the benefits created by digital consultations and how such outcomes might be measured and valued.

In conclusion, this study identified mechanisms through which digital consulting can lead to improved efficiencies for both clinics and patients. Qualitatively, digital consulting has positive potential economic impacts for NHS, such as preventing adverse events and improving efficiency and patient well-being despite increased staff workload. This study may be regarded as a preliminary study to inform the design of future economic evaluations and service implementation plans in this area.

## Appendix

Multimedia Appendix 1 This is a health economics questionnaire.

## Abbreviations

IQR: interquartile range
LYNC: Long-term conditions Young people Networked Communication
NHS: National Health Service
SMS: short message service
UK: United Kingdom
