# Supplementary material for: Benefits and Costs of Digital Consulting in Clinics Serving Young People With Long-Term Conditions: Mixed-Methods Approach
Source: JMIR Med Inform. 2018 Oct 30;6(4):e48. doi: 10.2196/medinform.9577 (PMC6234335; doi:10.2196/medinform.9577)
Supplement: Multimedia Appendix 1 [file medinform_v6i4e48_app1.pdf]

Appendix 1 : health economics questionnaire

Health Economics questions

1. How much time do you spend per day, on average, using each of the methods of digital clinical communication listed below **directly with patients (excluding communication about patients with colleagues)?** For each method, please place a tick in the box corresponding to your best estimate. If this is >60 minutes, please provide an approximate duration.

	**rarely/never**	**15min**	**30min**	**45min**	**60min**	**more than 60 min (please provide time)**
**Email**						(_ _ _ min)
**Text**						(_ _ _ min)
**Social Media (e.g. Facebook, Twittter**						(_ _ _ min)
**Mobile phone**						(_ _ _ min)
**Other (please specify**						(_ _ _ min)

2. Please could you indicate what equipment you use for digital clinical communication **directly with patients (excluding communication about patients with colleagues)**, and who provides it :

(please tick boxes below)

	Don't use	Provided by employer	Purchased myself
Mobile phone			
Tablet			
Laptop			
Other (please specify):			

3. a) Could you please tell me your grade? ………………

b) How many hours do you work a day?   (_ hour)
c) How many days do you work a week? (_ day)
